# Vascularization of the dorsal root ganglia and peripheral nerve of the mouse: Implications for chemical-induced peripheral sensory neuropathies

**DOI:** 10.1186/1744-8069-4-10

**Published:** 2008-03-19

**Authors:** Juan M Jimenez-Andrade, Monica B Herrera, Joseph R Ghilardi, Marina Vardanyan, Ohannes K Melemedjian, Patrick W Mantyh

**Affiliations:** 1 Department of Pharmacology, College of Medicine, University of Arizona, Tucson, AZ 85724, USA; 2 Research Service, VA Medical Center, Minneapolis, MN 55417, USA

## Abstract

Although a variety of industrial chemicals, as well as several chemotherapeutic agents used to treat cancer or HIV, preferentially induce a peripheral sensory neuropathy what remains unclear is why these agents induce a sensory vs. a motor or mixed neuropathy. Previous studies have shown that the endothelial cells that vascularize the dorsal root ganglion (DRG), which houses the primary afferent sensory neurons, are unique in that they have large fenestrations and are permeable to a variety of low and high molecular weight agents. In the present report we used whole-mount preparations, immunohistochemistry, and confocal laser scanning microscopy to show that the cell body-rich area of the L4 mouse DRG has a 7 fold higher density of CD31+ capillaries than cell fiber rich area of the DRG or the distal or proximal aspect of the sciatic nerve. This dense vascularization, coupled with the high permeability of these capillaries, may synergistically contribute, and in part explain, why many potentially neurotoxic agents preferentially accumulate and injure cells within the DRG. Currently, cancer survivors and HIV patients constitute the largest and most rapidly expanding groups that have chemically induced peripheral sensory neuropathy. Understanding the unique aspects of the vascularization of the DRG and closing the endothelial fenestrations of the rich vascular bed of capillaries that vascularize the DRG before intravenous administration of anti-neoplastic or anti-HIV therapies, may offer a mechanism based approach to attenuate these chemically induced peripheral neuropathies in these patients.

## Background

Previous reports in both humans with accidental exposure, and in experimental animals, have shown that a variety of industrial agents and heavy metals produce a predominant sensory neuropathy. This list includes cloramphenicol [1], disulfiram [1], nitrofurantoin [1], thalidomide [2], adriamycin [3], chlorobiphenyl [4], chlorodinitrobenzene [5], dinitrobenzene [5], clioquinol [2], arsenic [6], cadmium [7] and methyl mercury [8]. It is still not completely understood why these agents induce primarily a sensory and not a motor peripheral neuropathy although it has been shown that the vascular supply to the dorsal root ganglia (DRG) is unique in that it is highly permeable to a variety of low and high molecular weight compounds and that agents such a cadmium and methylmercury preferentially accumulate in the DRG [9,10].

Peripheral neuropathy is also a major side effect of many commonly used anti-neoplastic agents including taxanes (eg, paclitaxel, docetaxel), vinca alkaloids, platinum-based compounds (eg, cisplatin and oxaliplatin) and the proteosome inhibitor bortezomib [11-14]. Interestingly, a similar, predominately peripheral neuropathy is also frequently observed in HIV patients receiving commonly utilized antiretroviral agents such as didanosine, zalcitabine, stavudine and indinavir [15-21]. This peripheral neuropathy is the *de facto *toxicity that limits the administration of many commonly used anti-neoplastic and anti-HIV agents [11-14,20,21]. This is significant as the current trend is towards more aggressive chemotherapy as is evident in recent studies demonstrating that in many chemotherapeutic regimens increased dose is associated with a clear increase in patient survival [13,22-24].

In the present report we quantitatively examined the density of the vascular supply of the cell body rich area (CBRA), the nerve fiber rich area (NFRA) of the lumbar (L4) DRG of the mouse and compared these to the vascular supply of the proximal and distal aspect of the sciatic nerve. To accomplish this we used immunohistochemical staining of CD31 (which is also known as platelet-endothelial cell adhesion molecule) in both whole-mount, and sectioned DRG and examined and quantified this staining using confocal microscopy, three dimensional reconstruction and quantitative histomorphometry.

## Results

In order to determine the CD31+ blood vessel density within the peripheral nervous system, we performed immunohistochemical analysis using an antibody raised against platelet endothelial cell adhesion molecule CD31. The antibody against CD31 has been used as a pan-endothelial marker and stains endothelial cells present in blood vessels [25]. CD31+ immunostaining has been reported to be weak or absent in endothelial cells of murine lymphatic vessels [26,27].

**Figure 1:**
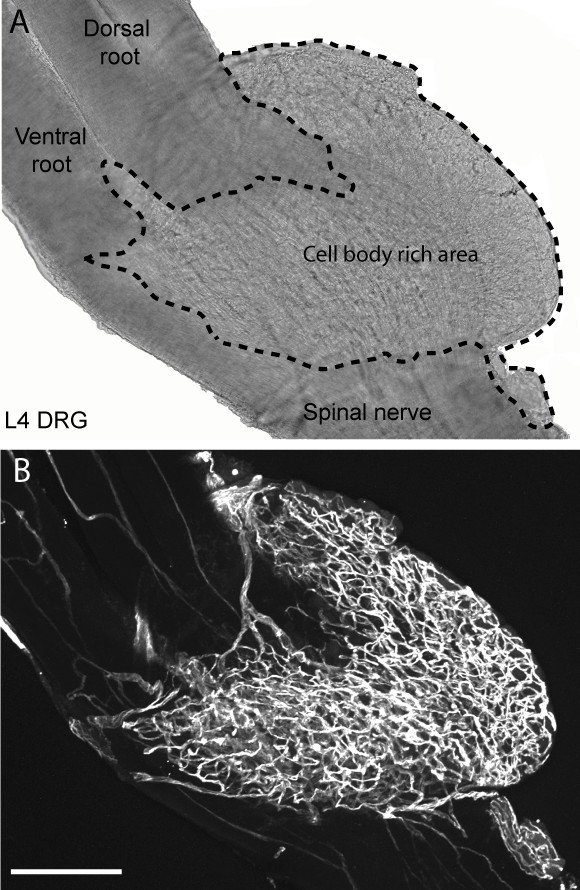
**Whole mount preparation showing the vascularization of the cell body rich area of the dorsal root ganglia (DRG) vs. the dorsal and ventral roots and sciatic nerve at L4 in the C3H mouse.** Bright-field photomicrograph of a whole-mount L4 DRG preparation for anatomical reference. Dashed line demarks the cell body-rich area from the sciatic nerve and dorsal and ventral spinal roots (A). Representative confocal micrograph of a mouse L4 DRG labeled with the endothelial cell marker CD31 showing the marked difference in the density of the vascular supply within the sensory ganglia as compared to the corresponding spinal nerve and dorsal root (B). This dense vascularization of the DRG along with the large fenestrations of the blood vessels in the DRG may partially explain why certain neurotoxics preferential accumulate in the DRG and produce a primarily sensory vs. motor neuropathy. The confocal image in (B) was assembled from 280 optical sections acquired at 0.5 μm z-plane intervals so that the total z stack is 140 μm-thick. Scale bar = 100 μm.

**Figure 2:**
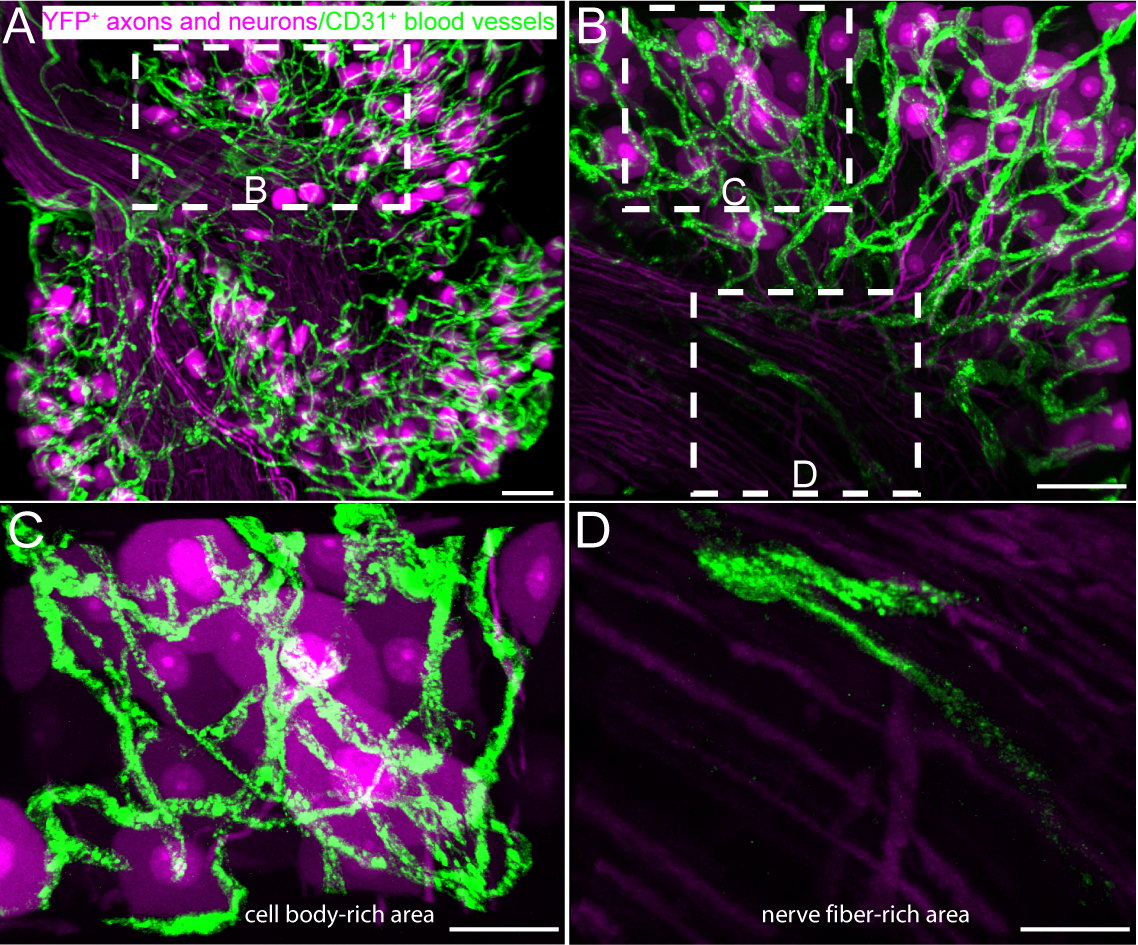
**The density of CD31+ vessels is site dependent within the mouse lumbar dorsal root ganglia (DRG).** Representative 3D reconstructed confocal images of L4 DRG whole mount preparation from thy1-YFP transgenic mice where the cell body and axons of sensory neurons constitutively express yellow fluorescent protein (YFP, pseudocolored violet). In contrast, the endothelial cells were immunohistochemically labeled with a marker of platelet endothelial cell adhesion molecule, CD31+ (green) (A, B). Note that a dense vascular plexus surrounds sensory neuron cell bodies within cell body-rich areas (C), whereas the nerve fiber-rich areas have a lower density of CD31+ vascular labeling (D). The confocal image in A-D were acquired at 0.5 μm z-plane intervals and the total z-plane for (A) 90 μm, (B) 60 μm and (C&D) 15 μm. Scale bar A-D = 50 μm.

Whole mount lumbar DRG with adjacent roots and spinal nerve attached were isolated from C3H/HeJ mice. While CD31+ blood vessels were present only sparsely within the endoneurium of the sciatic nerve, a dense network of CD31+ blood vessels was observed within the L4 DRG (Figure 1). In order to elucidate the association of blood vessels with cell bodies and axons of the sensory neurons within the DRG, CD31 immunohistochemical analysis on whole-mount preparations was conducted in L4 DRG of transgenic C57/B6 mice which constitutively express yellow fluorescence protein (YFP) in axons and cell bodies of sensory neurons (Fig 2A) [28,29]. YFP immunofluorescence was readily visible in cell bodies and axons of sensory neurons without signal amplification (Fig 2A). The 3D reconstructions of the confocal scans show the cell body-rich area (CBRA) is vascularized by an extensive network of CD31+ blood vessels that encapsulate and encircle the cell body of the YFP-expressing sensory neurons (Fig 2B,C). In contrast, the nerve fiber-rich area (NFRA) of the DRG contains relatively few CD31+ blood vessels (Fig 2B,D), and when present, run parallel to the bundles of nerve fibers.

**Figure 3:**
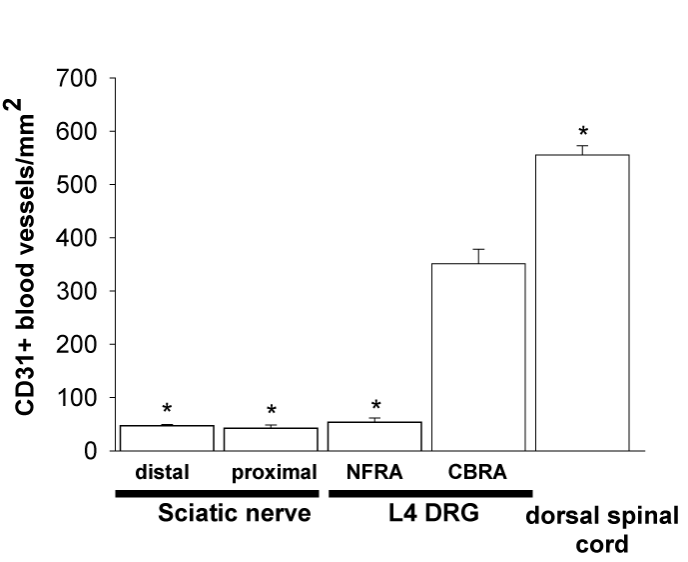
**CD31^+ ^blood vessel density is significantly higher in cell body-rich areas (CBRA) as compared to nerve fiber-rich areas (NFRA) of the L4 dorsal root ganglia (DRG) and distal and proximal sciatic nerve of the C3H mouse.** Regional differences in blood vessel density were determined by quantifying the CD31^+ ^blood vessels/mm^2 ^in 15 μm cut sections of the L4 DRG and attached nerve roots. Each bar of the histograms represents the mean +/- SEM of at least 4 mice and * indicates a difference of p < 0.05 vs CBRA of the L4 DRG.

To determine these regional differences in the density of CD31+ blood vessels within L4 DRG, we quantified the CD31+ blood vessels in CBRA and NFRA (Fig 3). The density of CD31+ blood vessels in CBRA (351 ± 27 CD31+ blood vessels/mm^2^) was significantly higher than that in NFRA (53 ± 8 CD31+ blood vessels/mm^2^). In order to compare the CD31+ blood vessel density of the CBRA to the peripheral nerves, we quantified CD31+ blood vessel density in two regions of the sciatic nerve. CD31+ blood vessels were observed mainly to run longitudinally along the endoneurium of the sciatic nerve (data not shown). No significant differences in the CD31+ blood vessel density were found between distal (47 ± 2 CD31+ blood vessels/mm^2^) and proximal (42 ± 6 CD31+ blood vessels/mm^2^) regions of the sciatic nerve (Fig 3). While the CD31+ blood vessel density in the sciatic nerve was similar in magnitude compared to that in NFRA, it was significantly lower as compared to that in CBRA (Fig 3). Finally, we quantified the CD31+ blood vessel density in the dorsal horn of the spinal cord, as this tissue is highly vascularized [30,31]. Results show that dorsal spinal cord is significantly higher than CBRA and has the highest blood vessel density (555 ± 17 CD31+ blood vessels/mm^2^) of the tissues evaluated in this study (Fig 3).

## Discussion

### The unique vascularization and permeability of the CBRA in the DRG

In the present study, we have shown that the CBRA of the DRG has approximately a seven fold higher density of the CD31+ blood vessels (which are mostly capillaries) than the NFRA of the DRG or the proximal and distal regions of the sciatic nerve. Interestingly, previous reports have shown similar striking regional differences in the expression of tight junction proteins and the presence of a functional blood-nerve barrier in the NFRA of the DRG but not in the CBRA of the rat DRG [32]. Previous studies have also shown that the blood vessels that vascularize the CBRA of the DRG have large fenestrations when compared to the peripheral nerves [33-35]. Thus, when large molecules such as albumin [32,36] or horseradish peroxidase [35,37] are injected into the rat tail vein these molecules remain in the vessels in the NFRA of the DRG and the vessels of the peripheral nerve but avidly leak out of the vessels that vascularize the CBRA of the DRG. Thus, the dense vascularization, lack of the full repertoire of tight junction proteins and the highly fenestrated blood vessels that are present in the CBRA of the DRG makes this area of the peripheral nerve uniquely open and accessible to a variety of low and high molecular weight agents [33]. Thus, even intravenous administration of fluorescently labeled Evans-blue albumin (MW 68,000) results in dense deposition of this tracer immediately adjacent to the plasma membrane of the soma of rat DRG neurons [32].

Together, these results suggest that a blood-nerve barrier is largely lacking in aspects of the peripheral nerve that house the cell bodies of sensory neurons. In addition to this relatively unrestricted access, there are high metabolic demands placed on the cell bodies [38] that support the long axons that innervate the most distal extremities. This may in part explain why intravenous administration of potentially neurotoxic agents such as methyl mercury show preferential access [33,39], accumulation [39-41] and toxicity [33,41-43] to sensory neurons when compared to motor neurons whose cell bodies are housed within the blood brain barrier within the ventral horn of the spinal cord.

### Chemical agents-induced peripheral neuropathy

Neurotoxicity is a major side effect of many commonly used anti-neoplastic and anti-HIV agents [11-19,44]. Previously, the dose limiting toxicity of many chemotherapeutic agents was hypersensitivity and neutropenia, but since the former can now be treated with antihistamines or steroids and the latter with granulocyte colony-stimulating factor [45], peripheral neuropathy is the *de facto *toxicity that limits the administration of many commonly used anti-neoplastic agents [11-14]. This is significant as the current trend in oncology is towards more aggressive chemotherapy as is evident in recent studies demonstrating that in many chemotherapeutic regimens increased dose is associated with a clear increase in patient survival [13,22-24].

In previous studies we showed that intravenous administration of paclitaxel, a commonly used chemotherapeutic agent, leads to the development of peripheral neuropathy characterized by injury of neuronal and non-neuronal cells in the DRG [46,47]. To define the location of the cells that showed the first sign of injury we administered this agent intravenously and then looked for the appearance of markers of cell injury in the rat DRG, peripheral nerve and spinal cord at 1, 3, 6 and 10 days following initial intravenous infusion of paclitaxel [42]. Using this strategy, it was found that at day 1 post-infusion there was a marked up-regulation of activated transcription factor-3 (ATF3), wich is a marker of cell injury/regeneration [48,49] in a subpopulation of large and small DRG neurons. In contrast, markers of cell injury in the proximal or distal aspects of the sciatic nerve were not observed until 10 days post-infusion of paclitaxel, suggesting that one of the initial sites of paclitaxel induced injury was in the CBRA of the DRG. Interestingly, by 10 days post-paclitaxel infusion there were clusters of satellite cells in the DRG which have been suggested to be a "tombstone" of dead sensory neuron cell bodies [47]. Similar "tombstones" have been observed in DRGs obtained at autopsies of cancer patients treated with cisplatin [50] and in AIDS patients treated with antiretroviral agents [15,51], which also frequently produce a predominantly sensory chemotherapy-induced peripheral neuropathy (CIPN) [16-18,20,21]. Together these preclinical and clinical studies demonstrate that there are signs of injury and death of the cell body of sensory neurons of rats and patients receiving chemotherapeutic drugs and that direct drug-induced injury to the cell body and its supporting cells may participate in the generation of CIPN.

### Implications and future directions

Defining the mechanisms and developing new therapies to attenuate or prevent therapy induced neuropathy in cancer and HIV patients are both needed and possible. Thus, in many cases these neuropathies are frequently the dose limiting toxicity that limits the ability of clinicians to pursue an aggressive chemotherapeutic regimen that will in large part determine the survival of the patient [52-54]. For example, in patients with metastatic colorectal cancer, oxaliplatin therapy offers clear dose-related benefits in promoting disease free survival [55]. However, in many cases optimal and aggressive dosing of oxaliplatin is limited by CIPN which results in reduction or cessation of chemotherapy resulting in decreased survival rates for these patients [52-54,56,57].

Therapy induced peripheral neuropathy presents a unique opportunity in neuropathobiology in that preemptive therapy can be commenced before the chemotherapeutic regimen begins. Previous studies in preclinical models of stroke and spinal cord injury have shown the significant advantages in preemptive versus post-injury administration of neuroprotective strategies [58]. However, the clinical reality in stroke and spinal cord injury is that neuroprotective therapies can usually only be initiated after the neuronal injury has occurred [58]. In contrast, anti-neoplastic or anti-HIV therapies offer a unique opportunity in that the precise time and extent of neuronal injury induced by the chemotherapeutic agent is known allowing preemptive trials to be conducted in both the preclinical and clinical setting.

Currently cancer and AIDS survivors constitute the largest and most rapidly expanding group of patients that have peripheral neuropathy and neuropathic pain [19,20,44,59]. Thus, developing a mechanism based understanding of how these chemotherapeutic agents induce this primarily peripheral sensory neuropathy and developing mechanism based therapies to prevent and/or attenuate these neuropathies offers a significant opportunity to impact both the survival and quality of life of these patients.

## Conclusion

The high density of the CD31+ capillaries in the cell body rich area of the DRG, coupled with relative lack of a functional nerve-blood barrier in these capillaries, may partly explain why many circulating neurotoxic agents preferentially accumulate and injure cells within the DRG and induce a sensory rather than a motor neuropathy. Understanding the unique aspects of the vascularization of the DRG and using this knowledge to modulate the permeability of the capillaries that vascularize the DRG, before intravenous administration of anti-neoplastic or anti-HIV therapies, may offer a mechanism based approach to blocking or attenuating chemically induced peripheral neuropathies in these patients.

## Methods

### Animal model

Experiments were performed on a total of 12 adult male C3H/HeJ (C3H) mice (Jackson Laboratories, Bar Harbor, Maine) and 12 adult C57 B6.Cg-Tg(Thy1-YFP)16Jrs/J mice (Jackson Laboratories, Bar Harbor, Maine), weighing 20–25 g. The transgenic mice constitutively express yellow fluorescent protein (YFP) in motor and sensory neurons under the control of neuron-specific regulatory elements from the Thy1 gene [28,29]. All procedures were approved by the Institutional Animal Care and Use Committee at the University of Minnesota.

### Preparation of tissue

Mice were sacrificed with CO_2 _and perfused intracardially with 20 ml of 0.1 M phosphate buffered saline (PBS) followed by 20 ml of 4% formaldehyde/12.5% picric acid solution in 0.1 M PBS. For whole-mount DRG preparations, the L3-L5 DRG of Thy1-YFP mice and C3H mice were harvested together with its adjacent spinal roots and nerve, post-fixed for 4 h in the perfusion fixative and then processed for immunohistochemistry (see below). For sectioned tissue, the lumbar (L3-L5) DRG, sciatic nerves, and lumbar spinal cord of C3H mice were removed, post-fixed for 4 h in the perfusion fixative, and cryoprotected for 24 h in 30% sucrose in 0.1 M PBS all at 4°C and then processed for immunohistochemistry. Longitudinal sciatic nerve sections (1.5 cm segment) were obtained at mid thigh level approximately 1.0 cm proximal to the trifurcation.

### Immunohistochemistry on whole-mount preparations

Qualitative analysis indicated no differences in the CD31+ blood vessel density in L3 to L5 DRG. Thus for quantification and presentation purposes only L4 DRG were used. The L4 DRG and attached nerves were incubated for 60 min at room temperature (RT) in a blocking solution of 3% normal donkey serum in PBS with 0.3% Triton-X 100 and then incubated overnight at RT in primary antisera against the platelet endothelial cell adhesion molecule CD31, a marker of endothelial cells present in blood vessels [26,27] (monoclonal rat anti-mouse CD31, 1:500, BD Pharmingen, San Diego, CA). YFP immunofluorescence in Thy1-YFP mice was readily visible under an epifluorescence microscope, thus no amplification of the signal was performed. Tissue was washed in PBS and incubated for 3 h at RT with a secondary antibody (Cy3 anti-rat; 1:600, Jackson ImmunoResearch, West Grove, PA). Finally, the DRG and attached nerves were washed 3 × 10 min in PBS, mounted on gelatin-coated slides, dried, dehydrated via an alcohol gradient (70, 90, and 100%), cleared in xylene, and coverslipped with DPX.

### Immunohistochemistry on sectioned tissue

Serial frozen sections of L4 DRG, lumbar spinal cord (coronal sections) and sciatic nerve (longitudinal sections) were cut at 15 μm on a cryostat and mounted onto gelatin-coated slides for immunohistochemical analysis. In this case, sectioned tissues were incubated overnight at RT in primary antisera against CD31. Secondary antibodies conjugated to various fluorescent markers (Cy2 1:200, Cy3 1:600; Jackson ImmunoResearch, West Grove, PA) were used and further immunohistochemical steps were performed as described above. To confirm the specificity of the primary antibody, controls included preabsorption with the corresponding synthetic peptide or omission of the primary antibody.

### Laser scanning confocal microscopy and three-dimensional reconstruction

Laser scanning confocal microscopy of the whole mount preparations was performed with a BX-61 microscope equipped with the Fluoview 1000 imaging software 5.0 (Olympus America Inc, Melville, NY). Confocal z-series at 0.5 μm intervals were acquired for each observation area and filtered by two-frame Kalman low-speed scans. YFP was excited by a laser wavelength of 488 nm, and emissions were detected using a 522-nm emission filter. Sequential acquisition mode was used to reduce bleed through. Z-series of the different experiments were imported from the Olympus Fluoview format to the Imaris Pro Software 5.7.0 (Bitplane AG, Zurich, CH). Image threshold and channel pseudocolors were adjusted, and 3D reconstruction was performed in the Surpass module. Images were cropped with Adobe Photoshop CS and thereafter assembled in Adobe Illustrator CS.

### Quantification of CD31+ blood vessels

Quantification of blood vessels was performed by determining the number of CD31 positive (+) blood vessels per unit area [30,60]. Only sectioned tissue from C3H mice was used to perform this quantification. Microvessel profiles were identified using criteria described by Weidner and colleagues [61] where the presence of a vessel lumen was not required to identify vessel profiles. Only CD31+ blood vessels that were 2–10 μm in diameter were counted and CD31+ branched blood vessels were counted as one vessel. The number of CD31+ blood vessels was determined in cell body-rich area (CBRA) and nerve fiber-rich area (NFRA) [32]. Images of the DRG sections were captured on an Olympus BX51 epifluorescent microscope fitted with an Olympus DP70 digital camera and areas of interest were determined using Image Pro Plus version 3.0 software (Media Cybernetics, Silver Spring, MD). The number of CD31+ blood vessels per outlined area from 4 sections per ganglion spaced 100 μm apart was averaged for each animal and results were expressed as total number of CD31+ blood vessels per unit area (mm^2^). Random 250 μm × 250 μm areas of the sciatic nerve and medial dorsal horn of the spinal cord were viewed at ×400 magnification and the number of CD31+ blood vessels was quantified. Distal (approximately 1.2–1.5 cm distance from tribifurcation) and proximal areas (approximately 2.2–2.5 cm distance from tribifurcation) of the sciatic nerve segment were used for quantification. Two areas were counted per section of 4 sections of spinal cord and sciatic nerve (proximal and distal) spaced 100 μm apart. Results are expressed as the mean number of CD31+ blood vessels per unit area (mm^2^) ± SEM. Statistical differences were determined using ANOVA followed by Tukey post hoc test. p < 0.05 was considered significant.

## Authors' contributions

JMJA and PWM participated in the design of the study. JMJA and JRG carried out the experiments and data analysis. JMJA, MH, MV, OKM, JRG and PWM participated in finalizing the manuscript.
